# Mental disorders in former street-working boys

**DOI:** 10.1007/s00787-023-02282-w

**Published:** 2023-08-20

**Authors:** Nezar Ismet Taib, Hans Arinell, Caisa Öster, Mia Ramklint

**Affiliations:** 1 Department of Medical Sciences, Child and Adolescent Psychiatry, Uppsala University Hospital, 751 85, Uppsala, Sweden; 2 Department of Medical Sciences, Psychiatry, Uppsala University Hospital, 751 85, Uppsala, Sweden

**Keywords:** Mental disorders, Continuity, Homotypic, Heterotypic, Street-working children, Follow-up study

## Abstract

The continuity of mental disorders in street-working children is rarely studied. This study therefore investigated homotypic continuity, recurrence of the same disorder, and heterotypic continuity, when a new disorder follows on the previous, of mental disorders from childhood to adulthood in street-working boys from Duhok City, Kurdistan Region of Iraq. Mental disorders were assessed by structured diagnostic interviews in 40 street-working boys in 2004–2005 and again in 2021, when the participants’ mean ages were 12.1 (SD 1.8) and 29.7 (SD 2.3), respectively. Mental disorders were common; 24 participants (60%) satisfied the criteria for at least one diagnosis at baseline and 28 (70%) at follow-up. Comorbidity increased from 1.2 (SD 1.4) disorders initially to 2.5 (SD 1.8) at follow-up. Only anxiety disorders showed homotypic continuity. Depressive disorders exhibited the greatest increase over time whereas externalizing disorders exhibited a decreasing tendency. The number of mental disorders in adulthood was related to the number of mental disorders in childhood but not to the number of childhood traumas experienced, having previously worked for more than two hours per day, having worked for over two years on the streets, or having at least one dead parent as a child. Parental ratings on the Child Behaviour Check List (CBCL) from childhood were also unrelated to the number of adult disorders. More longitudinal studies with bigger samples of both genders are needed to fully evaluate the continuity of mental disorders in street-working children and to determine whether the number of mental disorders in childhood is a stronger predictor of being mentally disordered in adult life than psychosocial risk factors or experiences of internalizing or externalizing symptoms in childhood.

## Introduction

The United Nations Children’s Fund (UNICEF) categorises children who work on the streets, or street children, according to their relationship to their family of origin. The first category consists of street-working children, who maintain regular ties with their families. Street-living children who maintain only weak relations with their families belong to the second category, while the third category consists of children from street families, i.e., children living on the streets with their families [1]. This study focuses on street-working children.

Previous studies have shown that the effects of street work on children’s health depend on the nature, type, and intensity of the work as well as the circumstances under which it is done [2]. The International Labour Organization (ILO) therefore permits children aged 12 years and above to perform light work that does not negatively affect their health and development or interfere with their education [3]. The term child labour thus refers to hard work that is harmful to the child, such as working before reaching 12 years of age, working many hours, performing heavy work, or labouring in dangerous workplaces [4, 5]. Child labour deprives children of their childhood, potential, and dignity, harms their physical and mental development, and interferes with their schooling [6]. Globally, it is estimated that around 160 million children are engaged in child labour and approximately 50% of these children are involved in hazardous work [7].

Evidence from previous studies suggest that street children experience multiple traumas including neglect, maltreatment, and psychological, physical, and sexual abuse [8, 9]). Moreover, they commonly report behavioural problems including problems with the police and monetary extortion [8]. Street children in the Middle East have a particularly high risk of experiencing violence, displacement, loss, torture, and other severe forms of traumas [10]. While street children reportedly have an elevated incidence of internalizing and externalizing symptoms, psychiatric diagnoses based on established diagnostic procedures have rarely been presented [11]. However, a study in street-working boys from Duhok in the Kurdistan region of Iraq where structured diagnostic interviews were used found that 61% had at least one mental disorder and 24% had two or more psychiatric diagnoses [12]. These street-working boys also reported more traumatic events than a matched control group consisting of school boys without experience of street work who were recruited some years later from the same region [13].

Anxiety/depression, delinquent behaviours, and aggressive behaviours in childhood are all generally considered core predictors of adult psychopathology [14]. Mental disorders show varying degrees of continuity from childhood to adulthood [15]; both homotypic continuity (i.e., recurrence of a disorder) and heterotypic continuity (i.e., a situation in which one disorder follows another) is commonly described, but heterotypic continuity is often related to specific pathways [16]. Anxiety disorders in childhood exhibit both homotypic and heterotypic continuity and increase the risk of other anxiety disorders as well as depressive disorders [17]. Adolescent depression often predicts young adult depression, and heterotypic continuity with anxiety disorders [16]. Externalizing disorders such as conduct disorders show homotypic continuity when it continues as antisocial personality disorder and heterotypic continuity with substance use disorders in adulthood [14]. There is a lack of studies exploring mental disorders in former street-working children, and the studies in this area that have been published often have methodological shortcomings due to the difficulty of gathering data from this group [18].

The high risk for impaired adult mental health for street-working children can be explained by exposure to high numbers of risk factors, such as poverty, trauma, school drop-outs, and early onset of mental disorders. Follow-up studies that include both social risk factors, traumas and early onset mental disorders are rare, if any.

This study is a long-term (16 years) follow-up of the previously mentioned study on street-working boys in Duhok that was conducted to evaluate the incidence of mental disorders in the now-adult participants and explore patterns of homotypic and heterotypic continuity of mental disorders from childhood to adulthood. Moreover, the relative influence of early onset disorders and risk factors for adult psychiatric outcome can be explored. We hypothesized that mental disorders would be common in the former street working children, and that there would be an increase in depressive disorders, with large homotypic continuity from childhood, but also new cases. We hypothesized that there would be both homotypic and heterotypic continuity of anxiety disorders, and we hypothesized that externalizing disorders would show homotypic continuity with externalizing disorders, even if there could be a change in specific diagnoses. Finally, we hypothesized that early onset of mental disorders mirrors individual vulnerability, also in those most socially burdened and traumatized, and therefore early onset mental disorders will explain adult mental disorder more than other social risk factors, i.e. daily working hours, years on the street, family economic status, parental education, parental loss, and traumatization.

## Method

### Study design

This is a 16-year follow-up of former street-working boys. The baseline study was a cross-sectional study about psychiatric morbidity among street-working children. The participants is now followed-up using the same diagnostic procedures.

### Participants

In 2004–2005, 100 street-working children were recruited as participants [12]. The participants constituted the total population of boys at a drop-in center for street-working children. The sample was a convenience sample due to the fact that there was no control over the selection of children for the drop-in center that was done by the police. At the time of inclusion, in 2004, seven members of the original cohort dropped out and were consecutively replaced by seven new members. These seven boys did not only drop out from the study, they also left the drop-in center. There is no information available about why, but these boys were very burdened by social problems. As soon as they disappeared, they were replaced by the first seven boys that were brought there by the police. All participants were boys because at the time no girls worked on the streets; their mean age was 12.1 years (SD 1.8). They were very poor and came from families whose average income per person was below half of the poverty line of Iraq at that time [19]. Most of their parents—95% of mothers and 90% of fathers—were illiterate. Fourteen boys had one dead parent and one had two dead parents. Seventy-six of the boys worked alone or with other children on the streets, while the remaining 24 worked with adults. The types of work they performed were most commonly selling goods in the streets or being shoe shiners [13].

In 2021, the former participants were 24–33 years old. In order to find them and invite them to participate in this follow-up study, we spoke to a social worker at the drop-in center from which the participants were initially recruited. This social worker was still in contact with 10 of the original participants, so he spoke to them and asked them to extend invitations to all the other former participants that they knew. All of the identified former participants (*n* = 42) were invited to visit the drop-in center, where they were informed about the study and invited for interviews after giving informed consent to participate in the study (*n* = 41). However, one of them did not return for his interview, so only 40 were interviewed. The mean age of the interviewees was 29.7 years (SD 2.3). They were as adults involved in variety of different occupations; 16 (40%) were workers on daily bases, 9 (22.5%) were soldiers or policemen, 6 (15%) had their own business (bakery, shop or hair salon), 3 (7.5%) were teachers, 3 (7.5%) were governmental employees, 2 (5%) were taxi drivers, and 1 (2.5%) was not working.

### Drop-out analysis

Table 1 shows the childhood characteristics of the participants and drop-outs. Except for mean age (*t* = 3.02, *p* < 0.01), the two groups did not differ significantly with respect to any of the measured factors, namely daily working hours (more or less than two hours), years spent working on the streets (1–2 years or 3–4 years), economic status of family of origin (low or average-good; this classification was based on the average family income in 2004–2005), father’s education (illiterate or at least primary school), mother’s education (illiterate or at least primary school), number of reported traumatic events, parent-reported emotional and behavioural problems or psychiatric disorders at baseline (none, 1, >  = 2).

**Table 1:** Description of childhood factors in participants (*n* = 40) and drop-outs (*n* = 60) in a follow-up study of former street-working boys conducted 16 years after the baseline study

Childhood factors in 2004/2005		Participants	Drop outs	*p*-value
		mean (SD)	mean (SD)	
Age		12.8 (2.1)	11.7 (1.5)	< 0.01
		*n* (%)	*n* (%)	
Working hours/day	1–2 h/day	28 (70)	42 (70)	1.00
	3–6 h/day	12 (30)	18 (30)	
Years working on the streets	1–2 years	30 (75)	40 (66.7)	0.37
	3–4 years	10 (25)	20 (33.3)	
Family’s economic status	Low	33 (82.5)	51 (85)	0.74
	Average-Good	7 (17.5)	9 (15)	
Fathers’ education	Illiterate	35 (87.5)	55 (91.7)	0.50
	At least primary school	5 (12.5)	5 (8.3)	
Mothers’ education	Illiterate	39 (97.5)	56 (93.3)	0.35
	At least primary school	1 (2.5)	4 (6.7)	
Parental loss	Parents alive	36 (90)	50 (83.3)	0.35
	Deceased parent(s)	4 (10)	10 (16.7)	
Number of traumatic events (HUTQ-C)		9 (5.6)	7.7 (6.8)	0.33
CBCL, Total Problem Scale		31.8 (18.7)	30.4 (17.4)	0.69
Mental disorders	No	16 (40)	23 (38.3)	0.86
	1	16 (40)	27 (45)	
	> = 2	8 (20)	10 (16.7)	

### Procedure

At both baseline and follow-up, each participant’s medical history was taken by the same person that afterwards conducted the diagnostic interview. At follow-up, but not at baseline, the structured diagnostic interview was followed by questions about their experiences of street work; the results obtained from the latter parts of the interviews have already been published [20].

## Instruments

### Baseline

#### MINI International Neuropsychiatric Interview for Children and Adolescents (MINI KID) version 6

The Mini-International Neuropsychiatric Interview for Children and Adolescents (MINI KID) is a structured diagnostic interview for DSM-IV and ICD-10 psychiatric disorders in children and adolescents that has shown good reliability and validity [21]. This interview was therefore used in 2004–2005, when the participants were interviewed by the first author (NT), who is a specialist in child and adolescent psychiatry with training in conducting MINI KID interviews. No formal translation of MINI KID was available at the time, so the English version was used and translated in real- time during the interviews. To increase the validity of the diagnoses and the similarity of the diagnostic procedure to that used clinically, the interviewers examined the interviewees’ medical history before conducting the MINI KID interviews. The medical history was taken, using a life-line, asking the child, or adult, about important life events as well as somatic and mental symptoms, and their relationships. The questions were open-ended. In addition to considering the respondents’ statements, the interviewer made clinical judgements by coding based on both the respondents’ answers and their medical histories. The interviewer also asked for examples whenever clarification was needed and instructed the adolescents to ask questions or request clarification if needed. NT had previously showed high inter-rater reliability (kappa = 0.97) performing the MINI KID this way. This inter-rater reliability was calculated based on ratings from NT and from another child and adolescent psychiatrist, based on her 12 video-taped MINI KID interviews. The interviews were made with the children without their parents, however the parents stayed in the next room and the child was instructed that if he wanted them to join, they could come at any time. In the end, no child asked for their parents.

### Child Behaviour Checklist (CBCL) 6–18

CBCL 6–18 is an instrument included in the Achenbach System of Empirically Based Assessment (ASEBA) for assessing competences and emotional and behavioural problems in 6 to 18-year-old children [22]. It consists of two broad-band scales (internalising and externalising), which are divided into eight subscales. This instrument was also used in 2004–2005. Because no formal translation of the CBCL into the Kurdish language exists, Thomas Achenbach’s permission to translate the questionnaire was sought and granted; the translation was performed by the first author, after which it was back-translated by a bilingual translator to verify the accuracy of the translation. The translated copy was field tested by the first author and a social worker by asking ten parents to evaluate the questions during interviews, allowing misunderstandings to be discussed as they arose. Minor cultural adaptations were performed as a result. The internal consistency of the Kurdish version of the CBCL was satisfactory (the Cronbach’s alpha values of the internalising and externalising scales were 0.77 and 0.81, respectively, and that for the total score was 0.92). Most of the parents of the studied children were illiterate, so all of the parents were interviewed by a social worker in 2004–2005 using the translated CBCL questions, which were read out verbatim to the parents. The CBCL also has 20 social competency items that measure children's involvement in activities (how much time they spend on sports, hobbies or games, and performance compared to same age peers; how active they are in organisations, clubs, or teams to which they belong; how well they carry out jobs or chores), social interaction patterns (how many close friends they have, how frequently they meet with friends, how well they get along with family members and other children, how independent they are when playing or working alone), and school performance (performance in academic subjects, academic or other problems in school). Scores for three narrow-band scales (Activities, Social, and School), and one broad-band scale representing Total Competence are provided [23].

### Harvard–Uppsala Trauma Questionnaire for Children (HUTQ-C)

The HUTQ-C was constructed to map out children’s experiences of traumatic events. It includes 30 items, 17 from the original adult scale and 13 extra items relevant to children living under difficult circumstances. These extra items consist of one old item (“lost or kidnapped”) that was split into two items together with 12 new items relating to experiences of road accidents, accidents at school, accidents during free time, technical accidents (for example, being stuck in an elevator), natural disasters, robbery, maltreatment/assault, bullying, being taken hostage, military mobilisation of a family member, and forced medical care, as well as terrifying hospital experiences. Some minor cultural and child-specific adaptations of the wordings were implemented—specifically, the item relating to forced separation from others was changed to forced separation from family members, and the item relating to rape or sexual abuse was changed to sexual abuse [24]. HUTQ-C was translated from English to Kurdish by the first author (NT) and then back-translated by a bilingual teacher to verify the accuracy of the translation. Discrepancies that emerged were then discussed until consensus was reached, after which the instrument was field tested. No additional changes were made after the field tests, which were conducted before the study began. The HUTQ-C was used in semi-structured interviews of children conducted in the presence of the child’s parent/caregiver by NT in 2004–2005. The parent/caregiver made comments after the child had commented, or before if the child requested support. A score of one point was assigned for every traumatic event experienced by the child.

### Follow-up

#### MINI International Neuropsychiatric Interview (MINI) version 7.0.2

The MINI is a brief structured interview for diagnosing the major mental disorders included in DSM-5 and ICD-10. Psychometric studies have shown that it has good reliability and validity [25]. Permission was obtained to use MINI English Version 7.0.2 in this follow-up study. No formal translation of MINI was available, so the English version was used and translated in real-time during the interviews. All interviews were performed by the same child psychiatrist who had performed the diagnostic interviews in childhood (NT). No formal inter-rater reliability test for this adult version of the interview was performed. MINI was used in the same way as MINI KID in 2004, meaning that each interview was performed only after taking the interviewees medical history, respondents were asked to provide examples whenever clarification was needed, and clinical judgements were made by coding on the basis of both the respondent’s medical history and their interview answers. The interviewer did not take part of the results from the base-line interview from 2004–2005, before meeting the participant.

#### Ethical considerations

Initial approval for the baseline study was obtained from the scientific research committee of the medical college at the University of Duhok in 2004. When the formal Ethics committee of Duhok University was established in 2013, their retrospective approval for the baseline study was sought and granted. Ethical approval for the follow-up study was granted by the Combined Ministry of Health/Duhok Directorate General of Health and Duhok University Research Ethical Committee (Dnr 13122020-6-12).

In the baseline study, informed written consent was obtained from every caregiver. For illiterate participants, the information was read out and they confirmed their acceptance by making a mark with their thumb. Participants were not compensated for taking part, and their data were stored confidentially. In the follow-up, the participants were given verbal and written information about the study, any concerns they had were addressed, and they were assured that their information would remain confidential. They then gave written informed consent. At both baseline and follow-up, participants who were diagnosed with mental disorders were referred to psychiatric services.

### Statistical analysis

Cronbach’s alpha was used to assess the internal consistency of the Kurdish versions of the CBCL and the HUTQ-C. Inter-rater reliability was measured with Cohen's Kappa. The psychiatric diagnoses were grouped into anxiety disorders, depressive disorders, and externalizing disorders. Diagnosed anxiety disorders included panic disorder, agoraphobia, social phobia, obsessive and compulsive disorder (OCD), posttraumatic stress disorder (PTSD), and generalized anxiety disorder (GAD) on both occasions, as well as specific phobia and separation anxiety disorders in the baseline study only. The only diagnosed depressive disorder was major depressive disorder; no cases of bipolar disorder were diagnosed. Diagnosed externalizing disorders included attention deficit hyperactivity disorder (ADHD), conduct disorder (CD), and oppositional defiant disorder (ODD) in the baseline study, and antisocial personality disorder in the follow-up study. Descriptive statistics were used to describe the characteristics of the participants and the prevalence of mental disorders. The chi-squared test was used to evaluate the significance of proportional differences between dichotomous variables. McNemar's test was used to explore differences in proportions over time. One-way ANOVA with Tukey’s post hoc test or the chi-squared test was used to compare social factors, HUTQ-C scores, and CBCL scores between groups with different numbers of adult diagnoses. Venn diagrams were used to visualize patterns of comorbidity, as well as changes in these patterns over time. Venn diagrams were generated using the R package "VennDiagram, version 1.7.1". All statistical analyses were performed using IBM SPSS Statistics for Windows, Version 26.0 (IBM Corporation, Armonk, NY). A significance threshold of p < 0.05 was applied in all analyses.

## Results

**Fig. 1:**
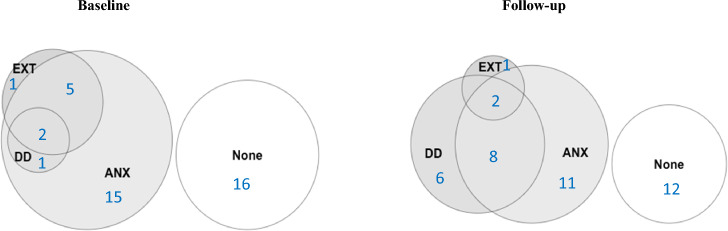
Venn diagrams showing the proportions and overlap of mental disorders in street-working boys (*N* = 40) at baseline and at follow-up after 16 years. *DD *depressive disorders, *EXT *externalising disorders, *ANX *anxiety disorders

The number of participants satisfying the criteria for any mental disorder was 24 (60%) at baseline and 28 (70%) at follow-up. Mental disorders at baseline and follow-up are presented in Table 2 and Fig. 1. No participants, either at baseline or at follow-up, were diagnosed with agoraphobia, bipolar disorder, alcohol or substance use disorders, any psychotic disorder, or any eating disorder.

**Table 2:** Mental disorders in street-working boys (*N* = 40) at baseline and follow-up after 16 years

	Baseline 2004/2005 *n* (%)	Follow-up 2021 *n* (%)
*Diagnoses assessed at both occasions*		
Major depressive disorder (MDD)	3 (7.5)	16 (40.0)
Panic disorder	4 (10.0)	6 (15.0)
Social phobia	2 (5.0)	7 (17.5)
Specific phobia	4 (10.0)	–
Generalized anxiety disorder (GAD)	3 (7.5)	3 (7.5)
Obsessive compulsive disorder (OCD)	3 (7.5)	8 (20)
Posttraumatic stress disorder (PTSD)	12 (30.0)	7 (17.5)
*Diagnoses assessed at one occasion*		
Conduct disorder (CD)	5 (12.5)	–
Oppositional defiant disorder (ODD)	0 (0)	–
Attention deficit hyperactivity disorder (ADHD)	4 (10.0)	–
Antisocial personality disorder	–	3 (7.5)
	**Mean (SD)**	**Mean (SD)**
**Number of diagnoses**	1.2 (1.4)	2.5 (1.8)

The proportion of participants diagnosed with any depressive disorder increased from 7.5% at baseline to 40% at follow-up (*p* = 0.002). The proportion diagnosed with any anxiety disorder and any externalizing disorder did not change significantly. Among those diagnosed with any anxiety disorder in childhood, 42.5% had an anxiety disorder in adulthood, (see Table 3 and Fig. 1).

**Table 3:** Prevalence of grouped mental disorders in street-working boys (N = 40) at baseline and follow-up after 16 years. McNemar’s test was used to explore differences in proportions over time and identify significantly changed proportions

Grouped mental disorders	Only at baseline *n* (%)	Both at baseline and follow-up *n* (%)	Only at follow-up *n* (%)	*p* value
Any anxiety disorder	6 (15.0)	17 (42.5)	4 (10.0)	0.75
Any depressive disorder	2 (5.0)	1 (2.5)	**15 (37.5)**	< 0.01
Any externalizing disorder	6 (15.0)	2 (5.0)	1 (2.5)	0.12

Table 4 relates various childhood factors to the occurrence of adult mental disorders. Participants with higher numbers of mental disorders in childhood also had higher numbers of mental disorders in adulthood. Neither social risk factors nor increased levels of symptoms (rated on the CBCL) were related to greater numbers of adult mental disorders.

**Table 4:** Comparisons of former street working boys (*N* = 40) with zero (*n* = 12), one (*n* = 15), and two or more (*n* = 13) adult mental disorders

Number of adult mental disorders in 2021 Factors in 2004/2005	0 disorders in adulthood *n* = 12	1 disorder in adulthood *n* = 15	> = 2 disorders in adulthood *n* = 13	*p* value
	Mean (SD)	Mean (SD)	Mean (SD)	
**Number of childhood mental disorders**	0.6 (0.9)	0.6 (0.6)	**1.8 (1.4)**	0.003
**Social factors**	*n* (%)	*n* (%)	*n* (%)	
Worked more than two hours a day on the streets	9 (75)	11 (73.3)	11 (84.6)	0.752
More than two years of work on the streets	5 (41.7)	3 (20)	2 (15.4)	0.270
At least one dead parent	1 (8.3)	1 (6.7)	2 (15.4)	0.726
**HUTQ-C**	Mean (SD)	Mean (SD)	Mean (SD)	
Number of experienced childhood traumas	7.6 (3.1)	10.7 (7.8)	8.3 (4.2)	0.318
**CBCL**	Mean (SD)	Mean (SD)	Mean (SD)	
Total competence scale	12.6 (4.5)	11.1 (3.2)	12.9 (3.6)	0.373
Internalizing problems scale	11.1 (6.2)	10.2 (5.8)	9.8 (6.4)	0.875
Externalizing problems scale	7.2 (5.2)	8.7 (5.1)	10.2 (9.3)	0.534
Total problems scale	33.3 (18.3)	30.1 (14.8)	32.5 (23.9)	0.890

## Discussion

This long-term follow-up study of former street-working boys from Duhok City in Iraq’s Kurdistan region explored the homotypic and heterotypic continuity of mental disorders from childhood to adult life, revealing that the participants who had more mental disorders in childhood were also found to have more mental disorders in adult life. The incidence of depressive disorders increased from childhood to adulthood, but that of anxiety disorders was unchanged. However, anxiety disorders were the most common disorders at both timepoints and generally exhibited a homotypic pattern of continuity. Externalizing disorders exhibited a non-significant decreasing trend over time. Comorbidity in childhood was related to comorbidity at follow-up. Neither measured social risk factors, nor higher scores on the CBCL scales were related to increased comorbidity at follow-up.

In a review of epidemiological studies, it was estimated that 25% of children experience a mental disorder in any given year [26], and a report produced by the World Health Organization states that up to 20% of children and adolescents worldwide suffer from a mental disorder [27]. The incidence of mental disorders in the group studied here was significantly higher than either of these values at the baseline. Two previous studies have used MINI KID to estimate the incidence of mental disorders in children living in societies similar to Iraqi Kurdistan (i.e., societies characterized by poverty, political instability, armed conflict, and large numbers of street children). However, these earlier studies reported much lower incidences of mental disorders—11% and 22% [28, 29]. Surprisingly, these figures are even lower than those expected for more privileged children (children protected by their parents and not working). Both of these earlier studies [28, 29] examined school children and children from the general population. Compared to two other studies which used similar diagnostic interviews with street children and child labourers, the incidence of mental disorders in this study was still higher [30, 31]. In one of these studies, which was conducted on 528 child laborers aged 5–15 years from Addis Ababa and which used the Diagnostic Interview for Children and Adolescents (DICA), the prevalence of any DSM-III-R childhood emotional and behavioural disorder was 20.1%. A cross-sectional study [31], using the Strength and Difficulties Questionnaire (SDQ), in 74 Zambian street children, 7–17 years old, found that 41% reported one mental health problem and 35% multiple problems. Despite being in the same age groups as these children, the street-working boys examined here are expected to be more severely burdened. The street working boys might also have discussed their experiences more openly with the interviewers because they were familiar with them and because all interviews were conducted in the presence of their caregivers in the next room, at a drop-in center that they frequently visited. These conditions maximized the likelihood that the vulnerable children participating in the study would truthfully report their symptoms and experiences.

In a systematic review of studies exploring the prevalence of mental disorders in adults, the 12-month prevalence was estimated to be 18% [32]; the highest prevalence reported in studies using the MINI diagnostic interview used in this work was 22% [29, 33, 34]. However, all these studies examined the general populations of secure and prosperous societies. Surprisingly, another systematic review of studies conducted in conflict-affected populations estimated the prevalence of mental disorders to be almost identical—22% [35]. These figures are much lower than the prevalence observed among the participants of this study despite clear similarities in the distributions of diagnoses [28, 29, 33, 34, 36]. The higher prevalence of mental disorders among the group studied here may be because the earlier studies targeted the general population rather than the most vulnerable individuals in society. Lay interviewers are generally expected to over-diagnose disorders, so the finding of a higher incidence of disorder in this work, where a child and adolescent psychiatrist performed the interviews, is unexpected. It is possible that the results obtained in this work include false positive reporting resulting from cultural coercion; the respondents may have adjusted their answers in an attempt to please a doctor who was trying to help them. Alternatively, the figures presented here could be accurate because the burdened participants dared to honestly report their experiences.

The patterns of homotypic and heterotypic continuity of disorders observed in this work were partly consistent with previous findings from different populations – for example, adult depression has previously been found to be common among individuals without mental disorders in childhood [36]. Although the expected heterotypic continuity between depressive disorders and anxiety disorders was not found, the heterotypic continuity between anxiety and depression was replicated, since all participants with adult depression had an anxiety disorder in childhood [17, 37]. The observed homotypic pattern of continuity in anxiety disorders is also consistent with previous reports [15–17].

A homotypic continuity was expected for externalizing disorders [38, 39] but was not observed. This could be because the baseline participants were street-working boys living with their families. They had not been pronounced delinquent; in fact, the majority had been loyal to their poor parents and had tried to support their families financially by working [20]. As a result, externalizing disorders were uncommon at the baseline. Moreover, some of the observed delinquent behaviours in childhood could have been due to environmental impacts from street life rather than internal pressure arising from oppositional traits.

The CBCL ratings were based on parental reports; the participants’ total competence, internalizing problems, externalizing problems, and total problems scores were rated by the social worker while interviewing the parents. Interestingly, the CBCL scores were surprisingly low when considered in relation to the diagnosed disorders. While we cannot say for certain what caused this apparent discrepancy between CBCL reports and the diagnostic evaluations, we suspect that some cultural factors may have affected both parental reports during CBCL assessments and children´s responses to the HUTQ. For example, no child reported sexual abuse but we know from the adult interviews [20] that many heard of and saw other children being abused but did not report this during childhood. We therefore believe that the diagnostic evaluation provides a more accurate reflection of the participants’ true circumstances than the parental reports used to obtain CBCL scores and some of the children’s reported traumas.

In accordance with the literature, almost all participants diagnosed with depression, both in childhood and adulthood, had a comorbid anxiety disorder [40, 41]. Comorbidity is common in mental disorders, and the total burden of disorders is known to have a negative impact on outcomes [42]. The finding that the number of mental disorders in childhood was related to that in adulthood is supported by a recent review of studies on continuity [43]. Childhood adversities such as maladaptive family functioning, child abuse, and neglect have been shown to be strong predictors of mental disorders in adulthood [44]. Several studies have examined risk and resiliency factors [44, 45] for mental disorders, but studies examining the longitudinal outcome of childhood risk factors together with childhood mental disorders in former street-working children are scarce. The main finding from this study is that early onset of mental disorders is probably the strongest risk factor for adult mental disorders in street-working children. In general, the onset of mental disorders is believed to be caused by number of adversities interacting with individual vulnerability [45]. It is possible that no significant relationship between adversity and adult disorders was detected only because the studied group was very homogenous in that all of its members were burdened by psychosocial risk factors. Consequently, individual vulnerability may have become the main factor differentiating those with and without disorders in this group.

### Strengths and limitations

This study has several limitations. First, it was difficult to locate all participants from the 2004–2005 interviews; there were more drop-outs than participants. Although an analysis of the drop-outs suggested that the adult participant group remained representative of the baseline group, the small sample size reduced the study’s statistical power. Additionally, the sample was very homogenous with respect to risk factors, making it difficult to evaluate the impact of specific risk factors. Major strengths of this study are that it is based on a long-term follow-up of a vulnerable group whose members are very difficult to find and follow, and that it is probably the first study looking at continuity of mental disorders rather than symptoms among former street-working children. Diagnoses retrieved from research studies must be questioned because it is harder to determine whether the mandatory criteria concerning functional impairment are fulfilled when those being diagnosed are not help-seeking. To overcome this difficulty, the diagnostic procedure was performed by a single person with expertise in child and adolescent psychiatry rather than a lay interviewer. Accordingly, it is a strength that mental disorders were assessed using gold standard diagnostic procedures whereas most previous studies in this field have relied on dimensional assessments of symptoms [46, 47]. The diagnostic procedure could be described as a compromised version of the Longitudinal Expert All Data (LEAD) procedure [48] because the interviewer used information from a preliminary medical history interview together with the information gathered through the structured diagnostic interview to assess all diagnostic criteria. However, even if NT did not take part of the base-line data before interviewing, he might have recalled some information while talking to the participant. That could have introduced some bias when interviewing at follow-up. However, there are 16 years between the examinations, and it is difficult to remember details, such as diagnostic information, for such a long period.

## Conclusion

The incidence of mental disorders in street-working children from Duhok, Kurdish region of Iraq, is high both in childhood and at adult follow-up. Anxiety disorders were the most common disorders in both cases, but depressive disorders exhibited the greatest increase from childhood to adulthood. Homotypic continuity was observed for anxiety disorders, together with heterotypic continuity from anxiety disorders to depressive disorders. More longitudinal studies with bigger samples of both genders are needed to further explore continuity of mental disorders in street-working children.

## Data Availability

Data supporting the findings of this study are available from the corresponding author [NT] on request.
